# Characterization of Local Structures of Confined Imidazolium Ionic Liquids in PVdF-co-HFP Matrices by High Pressure Infrared Spectroscopy

**DOI:** 10.3390/nano10101973

**Published:** 2020-10-05

**Authors:** Teng-Hui Wang, Ming-Siou Wu, Hai-Chou Chang

**Affiliations:** Department of Chemistry, National Dong Hwa University, Shoufeng, Hualien 974, Taiwan; 810712101@gms.ndhu.edu.tw (T.-H.W.); 610712011@gms.ndhu.edu.tw (M.-S.W.)

**Keywords:** poly(vinylidene fluoride-co-hexafluoropropylene) (PVdF-co-HFP), high-pressure, infrared spectroscopy, ionic liquids (ILs)

## Abstract

The nanoscale ion ordering of ionic liquids at confined interfaces under high pressures was investigated in this study. 1-Hexyl-3-methylimidazolium bis(trifluoromethylsulfonyl)imide ([HMIM][NTf_2_])/poly(vinylidene fluoride-co-hexafluoropropylene) (PVdF-co-HFP) and 1-ethyl-3-methylimidazolium bis(trifluoromethylsulfonyl)imide ([EMIM][NTf_2_])/PVdF-co-HFP were prepared and characterized by using high-pressure infrared spectroscopy. Under ambient pressure, imidazolium C^2^–H and C^4,5^–H absorptions were blue-shifted in frequency due to the presence of PVdF-co-HFP. However, the absorption of anionic ν_a_ SO_2_ did not reveal any significant shifts in frequency upon dilution by PVdF-co-HFP. The experimental results suggest that PVdF-co-HFP disturbs the local structures of the imidazolium C–H groups instead of the anionic SO_2_ groups. The frequency shifts of C^4,5^–H became dramatic for the mixtures at high pressures. These results suggest that pressure-enhanced ionic liquid–polymer interactions may play an appreciable role in IL-PVdF-co-HFP systems under high pressures. The pressure-induced blue-shifts due to the PVdF-co-HFP additions were more obvious for the [HMIM][NTf_2_] mixtures than for [EMIM][NTf_2_] mixtures.

## 1. Introduction

The high safety requirements of lithium-ion batteries in mobile devices such as smartphones and other flexible electronic devices such as smartphones and other flexible electronic devices have necessitated the development of noncombustible gel-like electrolytes [1,2,3,4]. Polymer electrolytes, comprising a combination of flexible semi-crystalline polymer matrices and electrolytes, can separate the cathode and anode in a battery, avoid short circuits, and maintain high ion conductivity. Poly(vinylidene fluoride-co-hexafluoropropylene) (PVdF-co-HFP) containing vinylidene fluoride (VdF) and hexafluoropropylene (HFP) moieties is a potential candidate for the matrix in polymer electrolytes [4,5,6,7,8,9]. The incorporation of the HFP unit in PVdF-co-HFP may increase the flexibility of the polymer due to the presence of low crystalline structures in the copolymers [5,8,9,10,11]. Researchers have shown that the low crystallinity of PVdF-co-HFP compared to the unmodified PVdF leads to a higher ionic conductivity in PVdF-co-HFP-based polymer electrolytes [5,6,7,8,10]. The crystalline phase of the polymers limits the migration of ions in Li-ion batteries. Flexibility of the polymers can be easily changed by adding plasticizers such as nanoparticles and ionic liquids (ILs) [1,5]. Researchers have discovered that PVdF-co-HFP and its mixtures hold promising applications as precursors of gas sensors [12] and can also be used in Li-ion batteries [1,4], dye-sensitized solar cells [13], and supercapacitors [2,6].

Based on the versatility of ionic liquids (ILs), there is the possibility of obtaining ILs with high ionic conductivities, large electrochemical windows, great thermal stability, gas adsorption, non-flammability, and low toxicity [14,15,16,17,18]. Some researchers have suggested that heterocyclic-based ILs such as alkyl-methyl-imidazolium ILs, are possible choices for polymer electrolytes due to the ease with which their physical properties can be modified [7,19]. However, the applications of imidazolium-based ILs in Li-ion systems are limited due to the reactivity of imidazolium C^2^–H (C–H at the C^2^ position of imidazolium cation). Due to their asymmetry, modifiable alkyl length, and the delocalized charge on the cation, the imidazolium cations and anions of the ILs may heterogeneously aggregate into nanostructures [20,21]. In other words, the cations and anions in the IL may associate together into clusters, resulting in the formation of quasi-crystal-like structures [20,21,22]. Additionally, previous studies have suggested that varying the alkyl chain length of the cations may affect the interactions between the IL ions, which can lead to changes in the associated structures [7,19,20]. There exists a debate about the interpretation of the IR spectra of 1-ethyl-3-methylimidazolium bis(trifluoromethylsulfonyl)imide ([EMIM][NTf_2_]) in the region of imidazolium C–H vibration [15]. The complicated C–H spectra features of [EMIM][NTf_2_] may result from micro-heterogeneity and Fermi-resonance interactions. The cluster model and hydrogen-bonding structures may play non-negligible roles in the ILs with [NTf_2_] anions [15].

Various studies on polymer electrolytes, comprising a polymer host and an IL guest, have revealed specific properties of ion migration in the polymers [1,2,3,5,6,13]. Additionally, it is important to understand the interactions between the polymer backbone and the IL ions in order to design suitable polymer electrolytes that can enhance their potential applications [2,3,23]. Liang et al. [24] concluded that the IL-polymer interactions mainly arise due to the associations between the positively charged ions (such as imidazolium, ammonium, and phosphonium) of the IL and the CF_2_ moiety in PVdF, rather than between the anions and polymer chains. In contrast, simulation studies have indicated that the hydrogen atoms of methylene groups present in the polymers (PVdF) are excellent nucleophilic sites and can interact with the anions ([BF4]) of ILs via electrostatic interactions. Natural bond orbital (NBO) [25] calculations suggest that the interaction energies between the ILs and PVdF do not depend on the alkyl chain length of the IL cation ([C_n_MIM]). Moreover, based on the results of the thermal properties, Jansen et al. [26] pointed out that the [EMIM][NTf_2_]/PVdF-co-HFP mixtures have a higher density than the linear interpolation of the value of the pure PVdF-co-HFP and pure [EMIM][NTf_2_]. This indicates an efficient packing and a high affinity between [EMIM][NTf_2_] and PVdF-co-HFP. The imidazolium-based ILs with [NTf2] anions and PVdF-co-HFP seem to have good compatibility to form blend membranes [7,26]. A thorough investigation of the interactions between polymers and IL ions (cations or anions) may provide information, which is pertinent to the future applications of polymer electrolytes. In this context, the precise mechanism of the interactions in polymer electrolytes is still a point of debate in the literature [24,25,26].

High-pressure infrared spectroscopy is a straightforward method to study the adjusted vibrational signals by forcing molecules to come close to each other. Modulating the molecular environments and non-covalent interactions by high pressures may provide special insights into the thermodynamically stable conformations of chemical systems [27,28,29,30,31]. This connotes that, under high-pressure conditions, the IL ions and the PVdF-co-HFP may aggregate at hydrostatic pressures. IR spectroscopy performed under high pressures may reveal whether it is the IL anions or the IL imidazolium cations that tend to associate with the polymers. In previous studies, IL cations have been known to form stable associations with deoxyribonucleic acid sodium salt (DNA) [27] under high pressures. Moreover, the results of previous studies imply that the IL hydrogen-bonding network or cluster structure may be disturbed on mixing additives such as porous silica [28], β-cyclodextrin [29], aluminum oxide [30], and mica [31] under high pressures.

## 2. Materials and Methods

The electrolyte samples were prepared using poly(vinylidene fluoride-co-hexafluoropropylene) (PVdF-co-HFP, average MW ~455,000, Sigma-Aldrich, St. Louis, MO, USA), 1-hexyl-3-methylimidazolium bis (trifluoromethylsulfonyl) imide ([HMIM][NTf_2_], ≥98%, Sigma-Aldrich, St. Louis, MO, USA), 1-ethyl-3-methylimidazolium bis (trifluoromethylsulfonyl) imide ([EMIM][NTf_2_], 99%, UniRegion Bio-Tech, Taoyuan, Taiwan), and N, N-dimethylformamide (DMF, ≥99.9%, Sigma-Aldrich, St. Louis, MO, USA). The water contents of [HMIM][NTf_2_] and [EMIM][NTf_2_] were determined to be 1.5 and 1.0 wt.%, respectively, by a moisture analyzer (MS-70, A&D Company). [HMIM][NTf_2_]/PVdF-co-HFP or [EMIM][NTf_2_]/PVdF-co-HFP mixtures were prepared with 10, 20, 30, 40, and 50 weight percentages of the IL and suitable amounts of DMF as the solvent. The solutions were then stirred at 70 °C for 30 min. The solvent (DMF) was removed under vacuum and the samples were kept in sunlight for at least one day. The mixtures were further dried at 155 °C with a moisture analyzer before all the spectral measurements. The removal of DMF was confirmed by the disappearance of DMF absorptions in the IR spectra.

High pressure (up to ~2 GPa) was generated using a diamond anvil cell (DAC) equipped with two type-IIa diamonds with a diamond culet size of 0.6 mm. IR measurements were performed using a Fourier-transform (FT) spectrophotometer (Spectrum RXI, Perkin-Elmer, Naperville, IL) equipped with a lithium tantalite detector. A 5-beam condenser was combined with the IR spectrophotometer to enhance the intensity of the passed infrared beam. To eliminate the absorption of the diamond anvils, the absorption spectra of the DAC were measured and subtracted from those of the samples. A 0.25 mm thick Inconel gasket with a 0.3 mm diameter hole was prepared (using a mechanical drill) to serve as the sample holder. To reduce the absorbance of the samples, transparent CaF_2_ crystals were placed into the sample holder and compressed before inserting the samples. A resolution of 4 cm^−1^ (data point resolution of 2 cm^−1^) and 1000 scans were chosen for the high-pressure measurements. Pressure calibration was performed following Wong’s method [32,33]. Spectral processing was performed with the software Origin. Gaussian or Lorentzian functions were applied for spectral deconvolution.

## 3. Results and Discussion

### 3.1. Ambient Pressure IR Study of [HMIM][NTf_2_]/PVdF-co-HEP System

**Figure 1:**
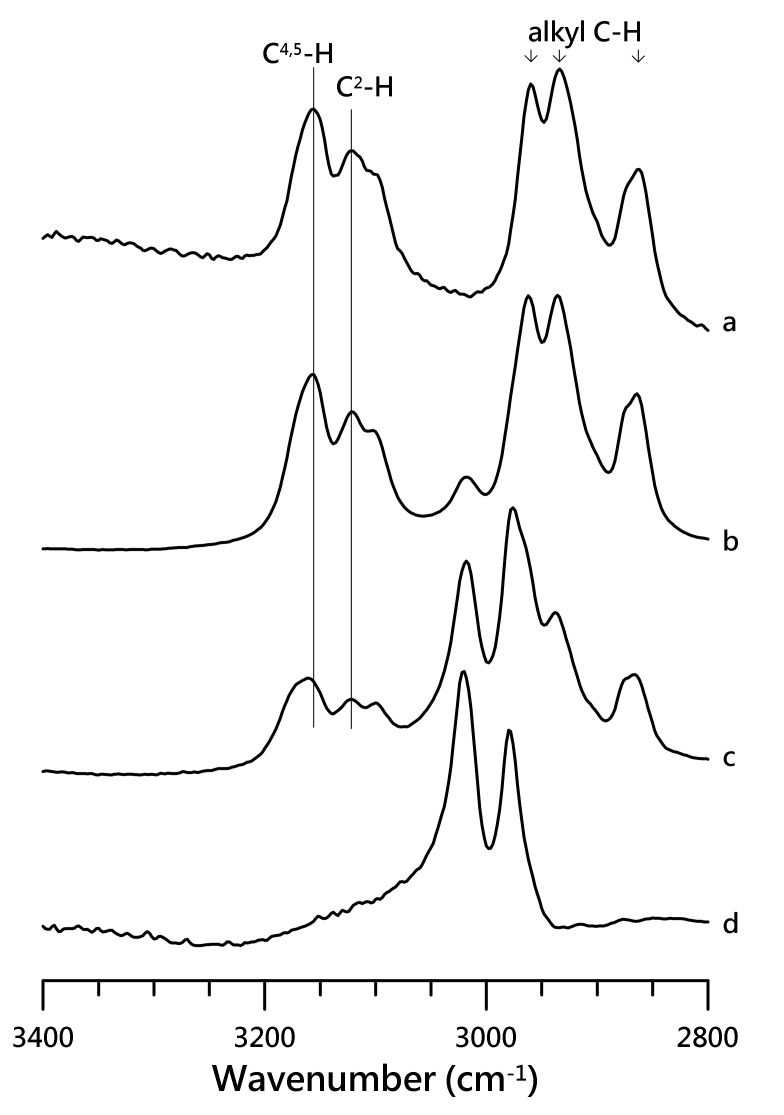
IR spectra of (**a**) pure [HMIM][NTf_2_], (**b**) mixture of PVdF-co-HFP with 50 wt.% of [HMIM][NTf_2_], (**c**) mixture of PVdF-co-HFP with 10 wt.% of [HMIM][NTf_2_], and (**d**) pure PVdF-co-HFP, recorded under ambient pressure.

Figure 1 presents the IR spectra of (a) pure [HMIM][NTf_2_], (b) mixture of PVdF-co-HFP with 50 wt.% of [HMIM][NTf_2_], (c) mixture of PVdF-co-HFP with 10 wt.% of [HMIM][NTf_2_], and (d) pure PVdF-co-HFP, which were recorded in the region of 3400–2800 cm^−1^ under ambient pressure. 

The IR spectrum of pure [HMIM][NTf_2_] contains vibrational absorptions at 2866, 2934, and 2959 cm^−1^ corresponding to the three alkyl C–H cations [27,28,29,30,31]. The IR spectrum of pure PVdF-co-HFP revealed two characteristic alkyl C–H peaks at 2979 and 3021 cm^−1^ [34]. The alkyl C–H peak of the cation at 2866 cm^−1^ in Figure 1a was very slightly shifted compared to the 2869 cm^−1^ peak obtained on mixing PVdF-co-HFP with the IL (Figure 1c). Nevertheless, the two other absorptions of the alkyl C–H cations at 2936 and 2962 cm^−1^, respectively, in Figure 1b,c overlap with the alkyl C–H peak of pure PVdF-co-HFP. The alkyl C–H absorption peak at 3018 cm^−1^ in the mixture of PVdF-co-HFP with 50 wt.% of [HMIM][NTf_2_] (Figure 1b) revealed a very small red shift compared to that of pure PVdF-co-HFP at 3021 cm^−1^ in Figure 1d. These observations imply that the alkyl C–H peaks of the cation and the polymer in the region between 2800 and 3075 cm^−1^ may not be sensitive enough to provide any valuable information. In the case of pure [HMIM][NTf_2_], the IR absorptions of the imidazolium C–H moieties are located at 3120 cm^−1^ (with a minor peak at ~3100 cm^−1^) and 3157 cm^−1^, corresponding to the C^2^–H and C^4,5^–H absorptions. However, the peaks for the imidazolium C^2^–H and C^4,5^–H groups shifted to 3121 and 3163 cm^−1^, respectively (Figure 1c), in the presence of PVdF-co-HFP.

**Figure 2:**
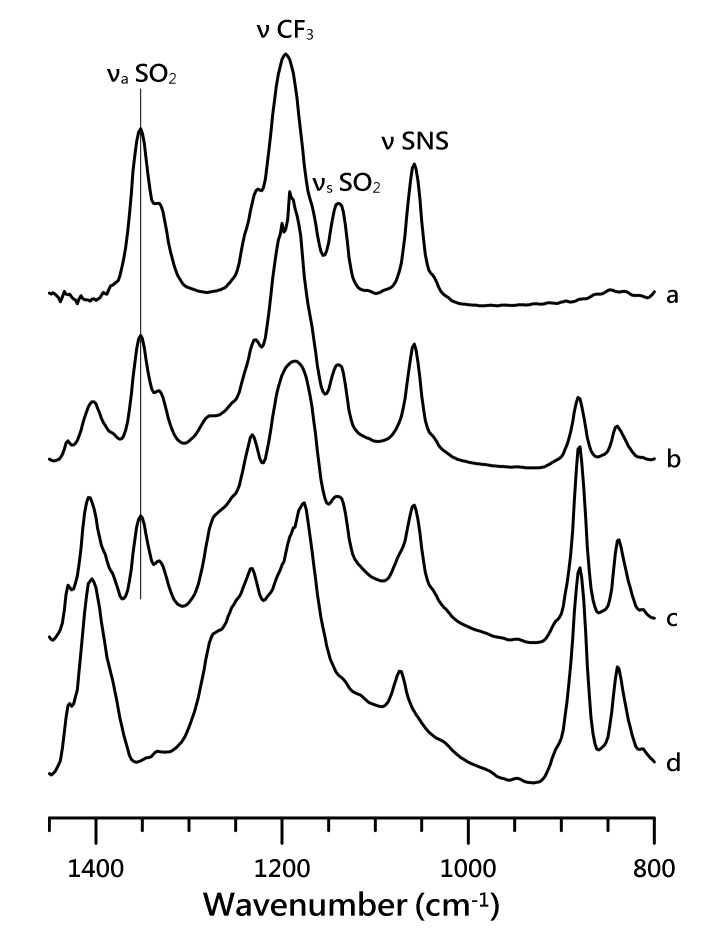
IR spectra of (**a**) pure [HMIM][NTf_2_], (**b**) mixture of PVdF-co-HFP with 50 wt.% of [HMIM][NTf_2_], (**c**) mixture of PVdF-co-HFP with 10 wt.% of [HMIM][NTf_2_], and (**d**) pure PVdF-co-HFP, recorded under ambient pressure.

Figure 2 illustrates the IR spectra of (a) pure [HMIM][NTf_2_], (b) mixture of PVdF-co-HFP with 50 wt.% of [HMIM][NTf_2_], (c) mixture of PVdF-co-HFP with 10 wt.% of [HMIM][NTf_2_], and (d) pure PVdF-co-HFP, which were recorded in the region of 800–1450 cm^−1^ under ambient pressure.

Figure 2a revealed four major vibrational absorptions at 1058 (ν SNS), 1140 (ν_s_ SO_2_), 1196 (ν CF_3_), and 1352 (ν_a_ SO_2_) cm^−1^, originating from the IL anion ([NTf_2_]^−^) [35,36]. Pure PVdF-co-HFP showed several characteristic polymer absorptions at 839, 881, 1074, 1179, 1233, and 1404 cm^−1^ [34]. The vibrational absorptions at 1196 and 1226 cm^−1^ for the CF_3_ moiety in [NTf2]^-^ overlap significantly with the CF_2_ absorptions of PVdF-co-HFP at 1179 and 1233 cm^−1^ for the mixtures (Figure 2b,c). Likewise, the spectral features of the IL anion at 1058 cm^−1^ (ν SNS) are also affected by the polymer absorption at 1074 cm^−1^, as seen in Figure 2b,c. The absorptions of ν_a_ SO_2_ at 1352 and 1334 (minor peak) cm^−1^ for pure [HMIM][NTf_2_] were slightly shifted to 1351 and 1333 cm^−1^ for the mixture of PVdF-co-HFP with 10 wt.% of [HMIM][NTf_2_]. Similarly, the ν_s_ SO_2_ band minimally blue-shifted (within 1 cm^−1^) to 1141 cm^−1^ (Figure 2c). The characteristic absorptions of PVDF-co-HFP in the mixture with 50 wt.% of [HMIM][NTf_2_] revealed almost negligible shifts to 839, 882, and 1403 cm^−1^ in comparison to the pure PVDF-co-HFP. These experimental results suggest that anionic SO_2_ groups and polymers may not significantly disturb each other. However, the results in Figure 1 indicate that the presence of PVdF-co-HFP disturbs the local structures of the imidazolium C–H groups in the IL cations [2].

**Figure 3:**
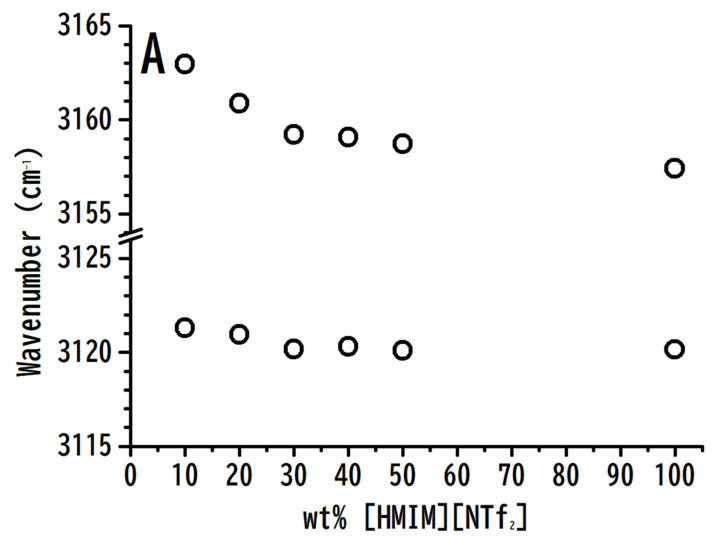

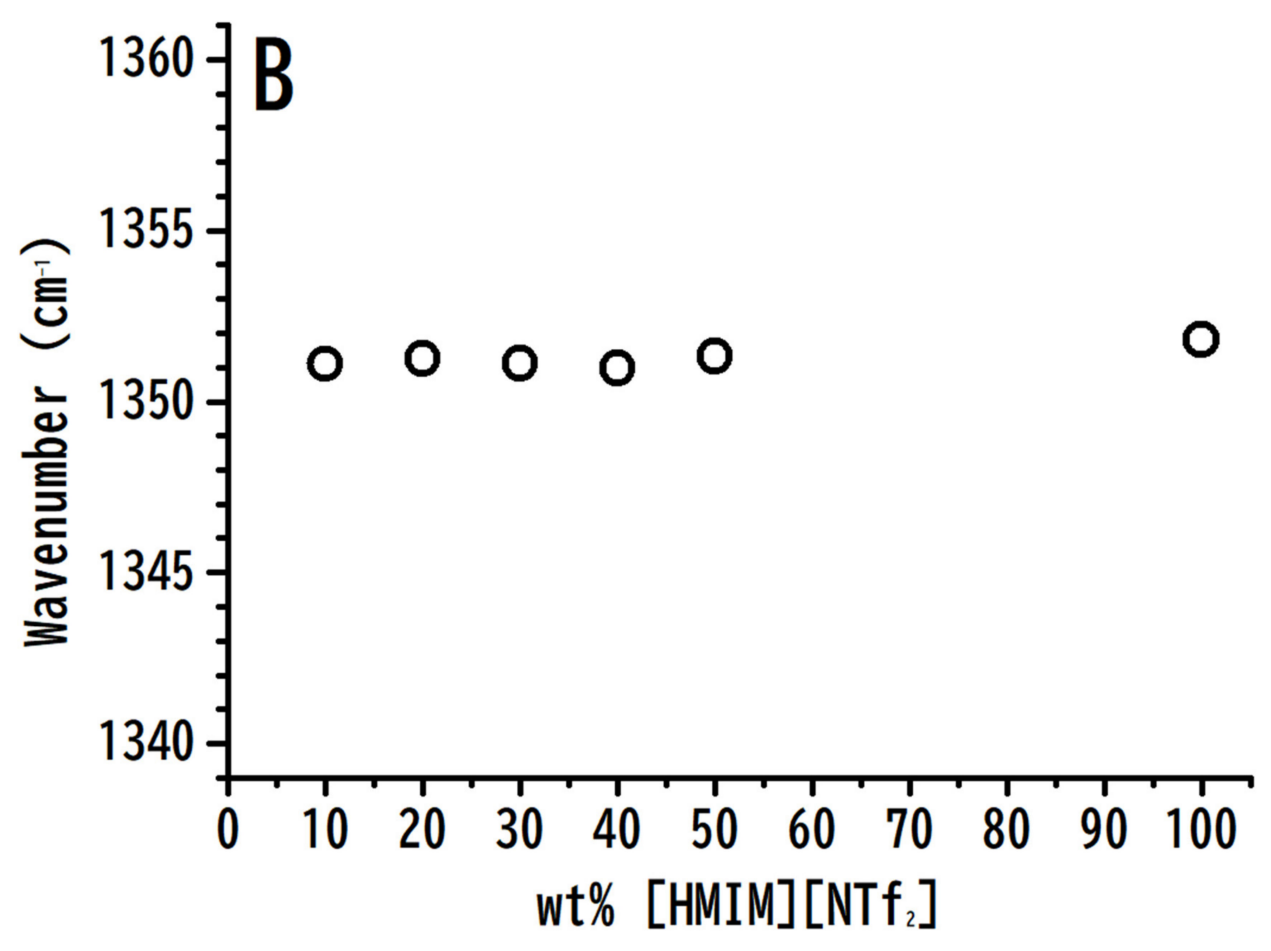
Concentration-dependence of the (**A**) imidazolium C^4,5^–H and C^2^–H, and (**B**) anionic ν_a_ SO_2_ stretching bands of [HMIM][NTf_2_]/PVdF-co-HFP mixtures versus the wt.% of [HMIM][NTf_2_].

Figure 3 depicts the concentration-dependence of (A) imidazolium C^4,5^–H and C^2^–H, and (B) anionic ν_a_ SO_2_ stretching frequencies versus the wt.% of [HMIM][NTf_2_]. The C^4,5^–H and C^2^–H absorption peaks of [HMIM][NTf_2_] showed a small frequency-shift with high concentrations of [HMIM][NTf_2_] (wt.% > 30%). In contrast, the C^4,5^–H and C^2^–H stretching frequencies were significantly blue-shifted with low concentrations of [HMIM][NTf_2_] (wt.% < 30%). Based on the experimental results (non-monotonic frequency-shift behavior) of Figure 3A, [HMIM][NTf_2_] does not seem to be homogeneously distributed within the polymeric matrix. Nanosegragation or domains of IL may exist especially for the mixtures with high concentrations of [HMIM][NTf_2_] (wt.% > 30%). The heterogeneous characteristic of ILs has attracted the attention of researchers. The non-monotonic frequency-shift behaviors revealed in Figure 3A may be related to the disturbance of inter-cluster and intra-cluster structures of ILs at high concentration and low concentration regions, respectively. The polymer may disturb the weaker inter-cluster interactions instead of the intra-cluster associations at high concentrations of [HMIM][NTf_2_] (wt.% > 30%). The anionic ν_a_ SO_2_ absorption peak in Figure 3B did not reveal any significant frequency shift with the change in concentrations of [HMIM][NTf_2_]. In conclusion, as revealed in Figure 3A, the imidazolium C^4,5^–H and C^2^–H in the ILs may be influenced by PVdF-co-HFP. Amongst these two moieties, the local structures of C^4,5^–H may be more easily disturbed by PVdF-co-HFP than those of the C^2^–H moiety.

### 3.2. Pressure-Dependent IR Study of [HMIM][NTf2]/PVdF-co-HEP System

**Figure 4:**
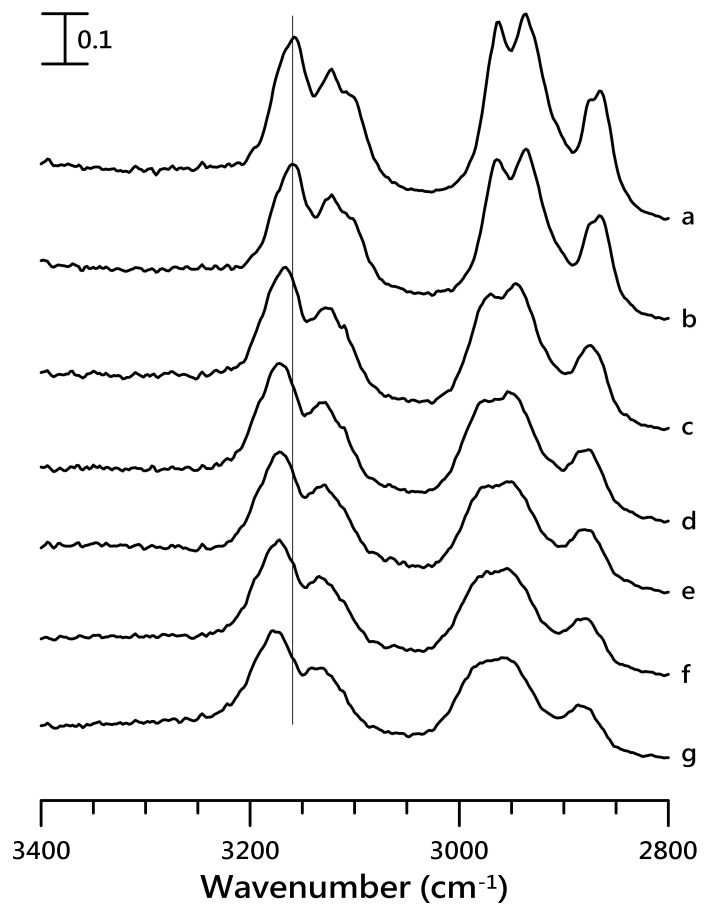
IR spectra of pure [HMIM][NTf_2_] obtained at (**a**) ambient pressure and (**b**) 0.4, (**c**) 0.7, (**d**) 1.1, (**e**) 1.5, (**f**) 1.8, and (**g**) 2.5 GPa.

Figure 4 shows the IR spectra of pure [HMIM][NTf_2_] obtained at (a) ambient pressure and (b) at pressures of 0.4, 0.7, 1.1, 1.5, 1.8, and 2.5 GPa in the region of 3400–2800 cm^−1^. On increasing the pressure from ambient condition to 0.4 GPa (Figure 4b), the imidazolium C–H and alkyl C–H spectral features did not reveal any notable changes. However, the peaks for the alkyl C–H and imidazolium C–H moieties revealed a blue-shift in the frequency and a broadening of the bandwidth upon further compression (Figure 4c–g). For instance, the peak for imidazolium C^4,5^–H at 3160 cm^−1^ (Figure 4b) shifted to 3168 cm^−1^ in Figure 4c.

**Figure 5:**
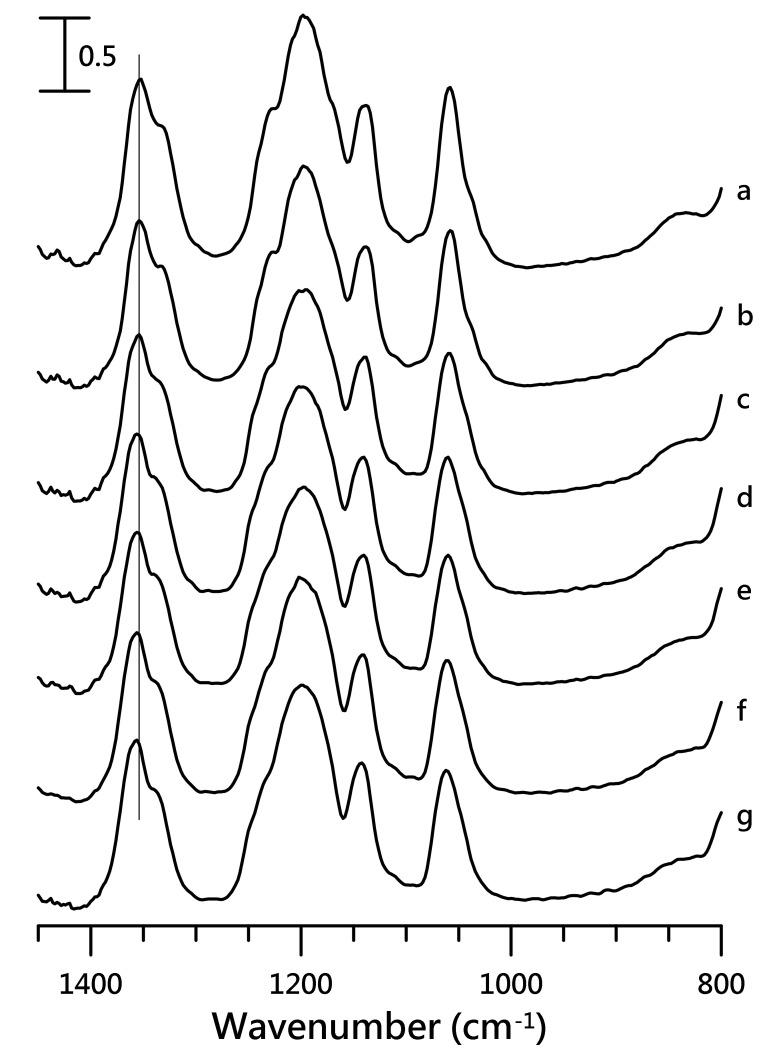
IR spectra of pure [HMIM][NTf_2_] obtained at (**a**) ambient pressure and (**b**) 0.4, (**c**) 0.7, (**d**) 1.1, (**e**) 1.5, (**f**) 1.8, and (**g**) 2.5 GPa.

The IR spectra of the anionic vibrations obtained at (a) ambient pressure and (b) 0.4, (c) 0.7, (d) 1.1, (e) 1.5, (f) 1.8, and (g) 2.5 GPa are displayed in Figure 5. For pure [HMIM][NTf_2_], ν_s_ SO_2_, ν CF_3_, and ν_a_ SO_2_ absorptions showed bandwidth changes and frequency shifts in the peaks as the pressure increased to 2.5 GPa, as shown in Figure 5. The ν_a_ SO_2_ absorption peak at 1352 cm^−1^ in the ambient condition was blue-shifted to 1357 cm^−1^ on applying a pressure of 2.5 GPa.

**Figure 6:**
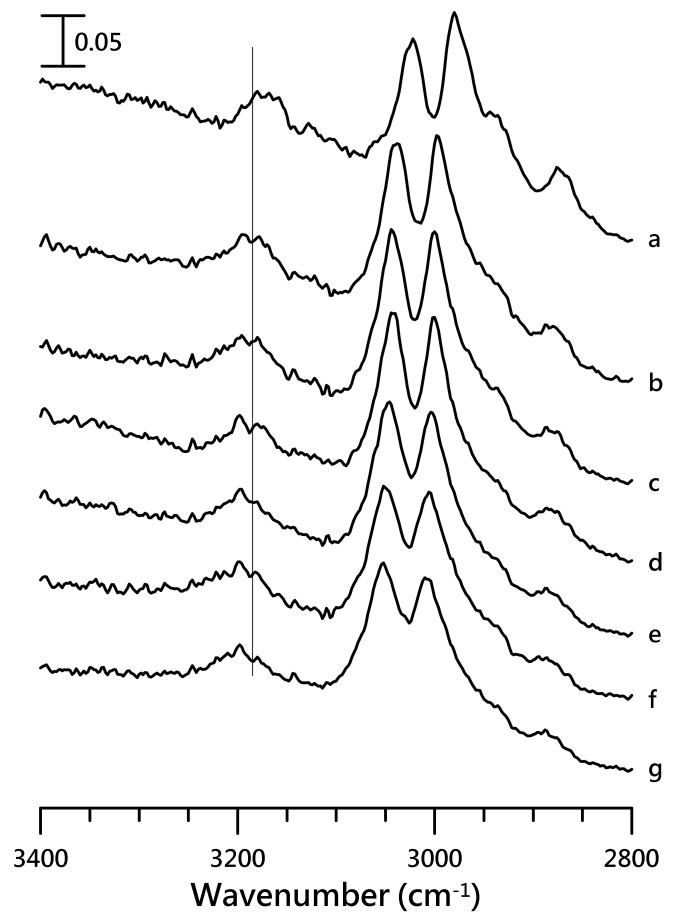
IR spectra of the [HMIM][NTF_2_]/PVdF-co-HFP mixture having 10 wt.% of [HMIM][NTf_2_], obtained at (**a**) ambient pressure and (**b**) 0.4, (**c**) 0.7, (**d**) 1.1, (**e**) 1.5, (**f**) 1.8, and (**g**) 2.5 GPa.

Figure 6 presents the IR spectra of the [HMIM][NTF_2_]/PVdF-co-HFP mixture having 10 wt.% of [HMIM][NTf_2_], which were recorded at (a) ambient pressure and (b) 0.4, (c) 0.7, (d) 1.1, (e) 1.5, (f) 1.8, and (g) 2.5 GPa. The alkyl C–H vibrational bands of PVdF-co-HFP (at 2979 and 3021 cm^−1^) displayed drastic blue shifts on applying a pressure 0.4 GPa (Figure 6b) and upon further compression, the peaks exhibited small frequency shifts (Figure 6c–g). The cation C–H absorption peaks were also significantly blue-shifted to 3193 and 2883 cm^−1^, corresponding to the imidazolium C^4,5^–H and alkyl C–H, respectively. IR spectra of the [HMIM][NTF_2_]/PVdF-co-HFP mixture with 10 wt.% of [HMIM][NTf_2_] for the SO_2_ stretching region are shown in Figure S1 in the Supplementary Materials.

**Figure 7:**
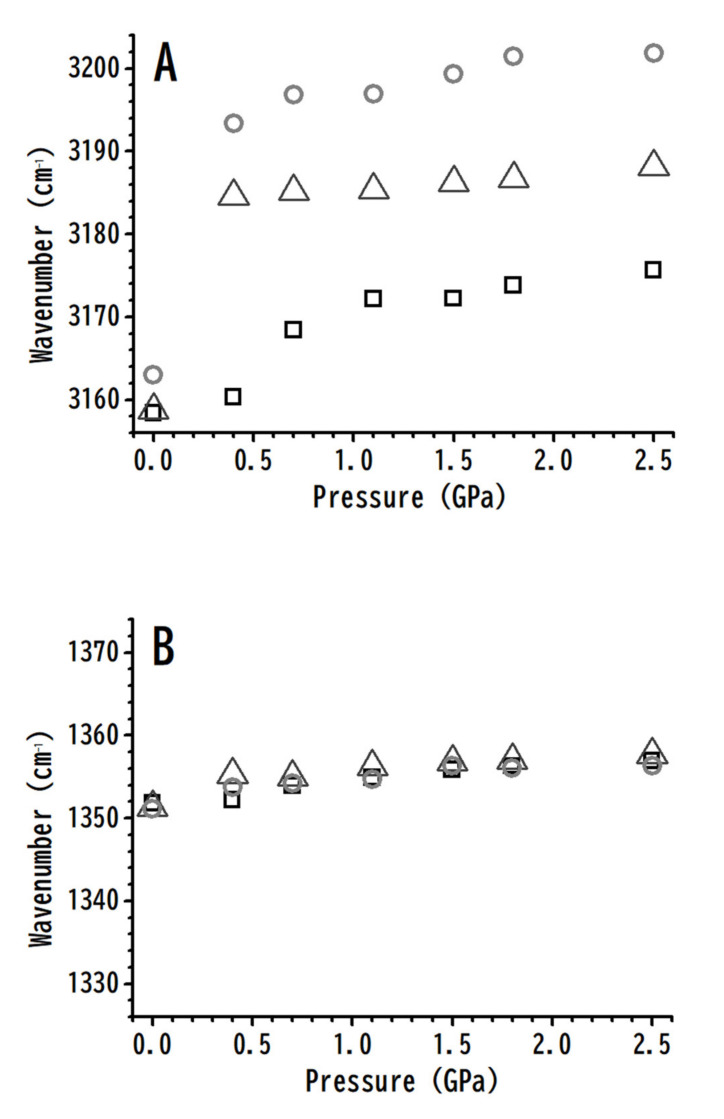
Pressure-dependence of the C–H stretching frequencies for (**A**) imidazolium C^4,5^–H and (**B**) anionic ν_a_ SO_2_ of pure [HMIM][NTf_2_] (squares) and the [HMIM][NTF_2_]/PVdF-co-HFP mixtures with 50 wt.% (triangles) and 10 wt.% (circles) of [HMIM][NTf_2_].

The pressure dependence of (A) imidazolium C^4,5^–H, and (B) anionic ν_a_ SO_2_ stretching frequencies with various amounts of [HMIM][NTf_2_] is shown in Figure 7. The C^4,5^–H band for the [HMIM][NTf_2_]/PVdF-co-HFP mixtures with 10 and 50 wt.% of [HMIM][NTf_2_] displays a dramatic blue-shift (pressure-induced phase transition) of 30 and 26 cm^−1^, respectively, on increasing the pressure to 0.4 GPa. Subtle frequency shifts were observed in the C^4,5^–H band upon further compression to 2.5 GPa. The C^4,5^–H absorption peak for pure [HMIM][NTf_2_] showed relatively smooth blue-shifts compared to those of the mixtures, at pressures below 0.7 GPa (Figure 7A). On increasing the pressure up to 0.7 GPa, the band was blue-shifted by 8 cm^−1^ in the case of pure [HMIM][NTf_2_]. We noticed that the frequency shifts in the C^4,5^–H band on varying the concentration of PVdF-co-HFP were more dramatic at high pressures (Figure 7A) than those at ambient pressure (Figure 3A). The reason for this might be that the local structures of C^4,5^–H are significantly perturbed by the polymer under higher pressures due to pressure-enhanced IL–polymer interactions. PVdF-co-HFP may intercalate into [HMIM][NTf_2_] clusters and interact with ILs at high pressures. Figure 7B indicates that the anionic ν_a_ SO_2_ stretching bands were not sensitive to the concentration variation of PVdF-co-HFP at various pressures. This suggests that the presence of PVdF-co-HFP does not disturb the local structures of the anionic SO_2_ groups under high pressures. Thus, the pressure-enhanced IL-polymer interactions seem to play a role in the IL-polymer systems under high pressures.

### 3.3. IR Study of [EMIM][NTf_2_]/PVdF-co-HEP System

**Figure 8:**
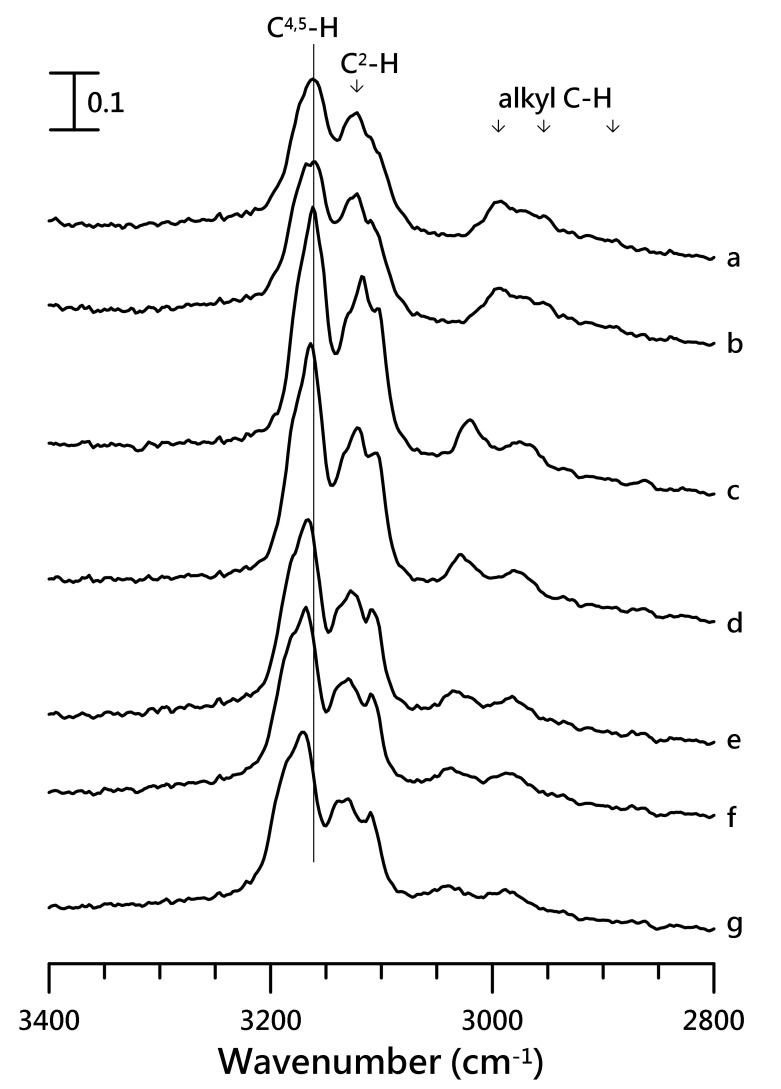
IR spectra of pure [EMIM][NTf_2_] obtained at (**a**) ambient pressure and (**b**) 0.4, (**c**) 0.7, (**d**) 1.1, (**e**) 1.5, (**f**) 1.8, and (**g**) 2.5 GPa.

To obtain a comprehensive view of the IL-polymer interactions, studies were carried out on ILs with short alkyl side chains, which is [EMIM][NTf_2_]. The IR spectrum with the cationic absorption of pure [EMIM][NTf_2_] was obtained at (a) ambient pressure and (b) 0.4, (c) 0.7, (d) 1.1, (e) 1.5, (f) 1.8, and (g) 2.5 GPa (Figure 8).

The C^4,5^–H peak of [EMIM][NTf_2_] at 3161 cm^−1^, obtained at ambient pressure, was blue-shifted to 3175 cm^−1^ on increasing the pressure to 2.5 GPa (Figure 8g). The C^4,5^–H and C^2^–H absorptions in Figure 8 revealed the changes in spectral features with pressure. Based on the results shown in Figure 8, it can be speculated that pressure may interfere with the cluster association of [EMIM][NTf_2_]. The pressure-induced phase transition or solidification may likely take place at a pressure of <0.7 GPa (see Figure 8c) for pure [EMIM][NTf_2_]. The peak narrowing observed at high pressures (*p* > 0.7 GPa) may suggest the formation of ordered structures. Different solidification pressures of ionic liquids for IR and Raman measurements have been reported in the literature [22,37,38] The different solidification pressures may originate from the various amounts of water in ILs [39] and the different experimental configurations inherent to IR and Raman techniques. It is instructive to note that the presence of water in ILs may significantly influence the overall phase behaviors. For the high-pressure IR measurements, Ca_2_F crystals were placed into the sample holder to avoid power saturation of the ionic liquid’s IR band. The CaF_2_ grains likely provide numerous nucleation sites that enable solidification of the ionic liquid. The addition of CaF_2_ is not required for high-pressure Raman measurements, and the ionic liquid is in contact with the smooth diamond surface, which present fewer nucleation sites. Thus, the phase transformation is accompanied by some degree of hysteresis in the Raman data, with the crystalline form persisting to pressures <1 GPa during decompression [38].

**Figure 9:**
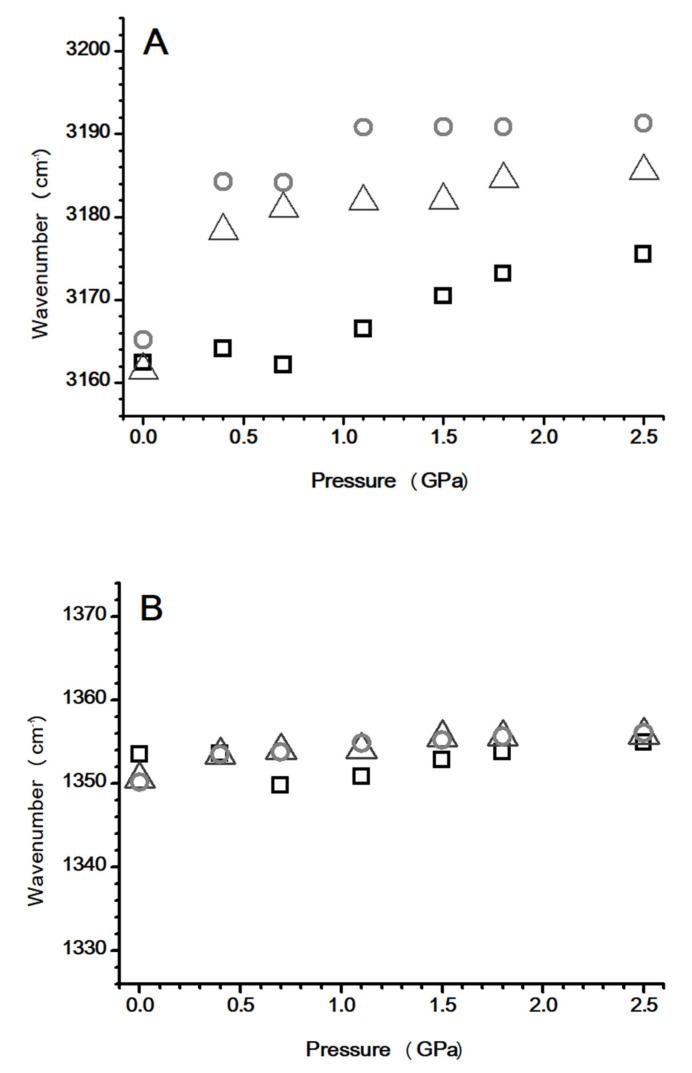
Pressure dependence of the C–H stretching frequencies for (**A**) imidazolium C^4,5^–H and (**B**) anionic ν_a_ SO_2_ of pure [EMIM][NTf_2_] (squares) and the [EMIM][NTF_2_]/PVdF-co-HFP mixtures with 50 wt.% (triangles) and 10 wt.% (circles) of [EMIM][NTf_2_].

The C^4,5^–H and ν_a_ SO_2_ stretching frequencies of [EMIM][NTf_2_]/PVdF-co-HFP mixtures with 10, 50 wt.% of [EMIM][NTf_2_], and pure [EMIM][NTf_2_] under various pressures are shown in Figure 9.

The C^4,5^–H band of pure [EMIM][NTf_2_] (Figure 9A) was blue-shifted on increasing the pressure from ambient condition to 0.4 GPa. The peak only slightly shifted on further increasing the pressure to 2.5 GPa. The C^4,5^–H absorption of [EMIM][NTf_2_]/PVdF-co-HFP mixtures with 50 and 10 wt.% of [EMIM][NTf_2_] also showed blue-shifts on increasing the pressure from ambient condition to 0.4 GPa. As shown in Figure 9A, the C^4,5^–H absorption band of pure [EMIM][NTf_2_] at 3161 cm^−1^ was slightly blue-shifted to 3165 cm^−1^ for the 10 wt.% mixture at ambient condition. Under a pressure of 2.5 GPa, the C^4,5^–H band was located at 3177 cm^−1^ and 3191 cm^−1^ for pure [EMIM][NTf_2_] and the 10 wt.% mixture, respectively. Hence, the frequency shifts (ν(10 wt.%) − ν(pure)) caused by the presence of PVdF-co-HFP increased from 4 cm^−1^ at ambient pressure to 14 cm^−1^ at 2.5 GPa. It may be possible that PVdF-co-HFP interjects into the IL cluster via pressure-enhanced IL–polymer interactions under high pressures. We note that the blue shifts due to the addition of PVdF-co-HFP under high pressures were more obvious for the [HMIM][NTf_2_] mixtures (Figure 7A) than those for the [EMIM][NTf_2_] mixtures (Figure 9A). These results indicate that the length of the alkyl side chain plays an appreciable role in the IL-PVdF-co-HFP interactions. As shown in Figure 9, the pressure-dependent anionic ν_a_ SO_2_ stretching frequencies were almost identical for the various concentrations of PVdF-co-HFP. The IR spectra of the [EMIM][NTF_2_]/PVdF-co-HFP mixture with 10 wt.% of [HMIM][NTf_2_] for the SO_2_ stretching region are shown in Figure S2 in the Supplementary Materials. The presence of polymers may not interfere with the anionic SO_2_ local structures under high pressures, as shown in Figure 9B, and Figure 7B for [EMIM][NTf_2_] and [HMIM][NTF_2_], respectively. By the way, the reversibility of IR spectra upon pressure-cycling was checked (see Figure S3 in the Supplementary Materials). In order to check that there were no obvious hysteretic behaviors, IR spectra with time under the pressure of 0.7 GPa were measured and are displayed in Figure S4 in the Supplementary Materials. To reduce the influence of water content [39] in this study, pressure-dependent IR spectra of pre-heated [EMIM][NTf_2_] (up to 155 °C) were obtained and are shown in Figure S5 in the Supplementary Materials.

**Figure 10:**
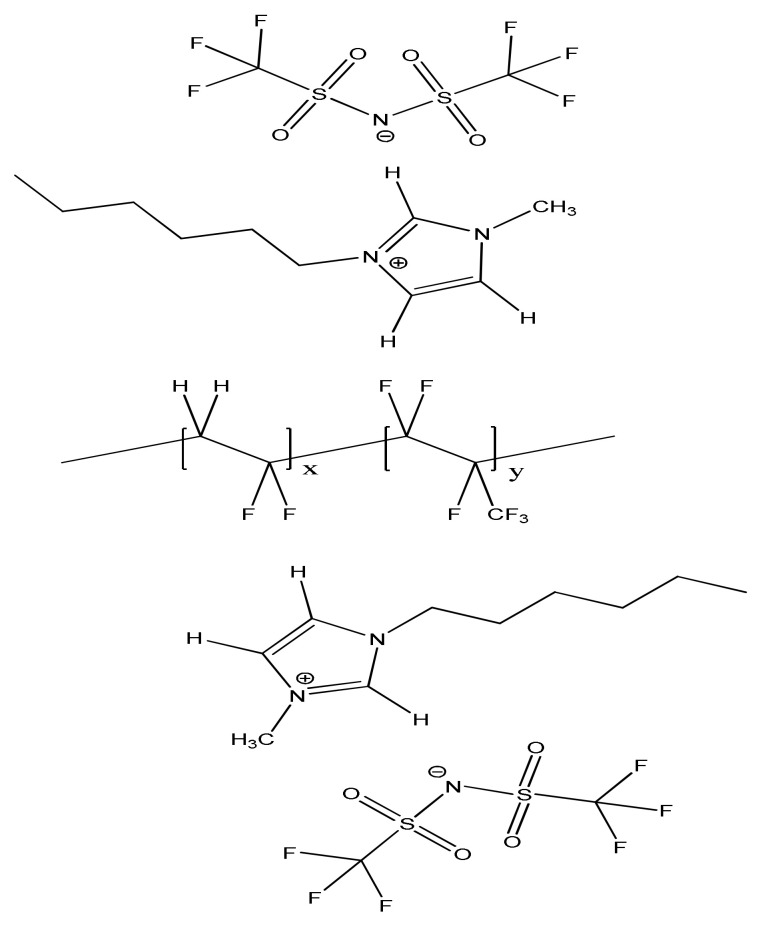
Illustration of the possible [HMIM][NTf_2_]-PVdF-co-HFP structures in the mixtures.

Figure 10 is a schematic illustration of the possible interactions between [HMIM][NTf_2_] and PVdF-co-HFP in the[HMIM][NTf_2_]-PVdF-co-HFP mixtures, The addition of PVdF-co-HFP may produce the interactions such as the C^4,5^–H–PVdF-co-HFP associations, while the local structures of the C^2^–H and anionic SO_2_ groups revealed remarkably less changes. Pressures used in this study ranged from ambient to several GPa; such pressures mainly change intermolecular distances and affect conformations. In fact, pressures in excess of 30 GPa are required to change the electronic structure of a molecule. In this study, we show that high pressure is a sensitive method to investigate how the local structure responds to pressure variation.

## 4. Conclusions

In this study, the local nanostructures of imidazolium IL-PVdF-co-HFP mixtures were investigated by using high-pressure infrared spectroscopy. The imidazolium C^4,5^–H vibration was blue-shifted non-monotonically on mixing the IL with PVdF-co-HFP under ambient pressure. The C^4,5^–H absorptions of the mixtures exhibited dramatic blue-shifts on increasing the pressure to values higher than 0.4 GPa. The results indicated that the local structures of imidazolium C^4,5^–H were significantly disturbed by the pressure-enhanced C^4,5^–H–polymer interactions. Nevertheless, the anionic ν_a_ SO_2_ absorption frequencies were almost identical for various pressures. The local structures of the anionic SO_2_ groups were not disturbed under high pressures. Due to the overlapping of absorptions from PVdF-co-HFP, the peaks from [NTf_2_] that we were able to identify and analyze were limited (only SO_2_). Thus, complementary techniques are needed to study the interactions and the role of the anions of these ILs in the IL/PVdF-co-HFP mixtures This study suggests that pressure-enhance IL–polymer interactions play a prominent role under high pressures.

## Supplementary Materials

The following are available online at https://www.mdpi.com/2079-4991/10/10/1973/s1, Figure S1: IR spectra of the [HMIM][NTF_2_]/PVdF-co-HFP mixture featuring 10 wt.% of [HMIM][NTf_2_] obtained at (a) ambient pressure and (b) 0.4, (c) 0.7, (d) 1.1, (e) 1.5, (f) 1.8, and (g) 2.5 GPa, Figure S2: IR spectra of the [EMIM][NTF2]/PVdF-co-HFP mixture featuring 10 wt.% of [EMIM][NTf2] obtained at (a) ambient pressure and (b) 0.4, (c) 0.7, (d) 1.1, (e) 1.5, (f) 1.8, and (g) 2.5 GPa, Figure S3: IR spectra of the [HMIM][NTF_2_]/PVdF-co-HFP mixture featuring 50 wt.% of [HMIM][NTf_2_] obtained at (a) ambient pressure, (b) 2.5 GPa, and (c) cycle back to ambient pressure, Figure S4: IR spectra of pure [EMIM][NTf_2_] obtained at the time of (a) 5 min (100 scans), (b) 1 h (1000 scans), (c) 2 h (1000 scans), and (d) 3 h (1000 scans) after the compression under the pressure of 0.7 GPa, Figure S5: IR spectra of pure [EMIM][NTf_2_] (pre-heated to 155 °C) obtained at (a) ambient pressure and (b) 0.4, (c) 0.7, (d) 1.1, (e) 1.5, (f) 1.8, and (g) 2.5 GPa at 25 °C.
