# Supplementary material for: Characterization of Local Structures of Confined Imidazolium Ionic Liquids in PVdF-co-HFP Matrices by High Pressure Infrared Spectroscopy
Source: Nanomaterials (Basel). 2020 Oct 5;10(10):1973. doi: 10.3390/nano10101973 (PMC7600376; doi:10.3390/nano10101973)
Supplement: Supplementary file 1 [file nanomaterials-10-01973-s001.pdf]

Supplementary Materials

Characterization of Local Structures of Confined Imidazolium Ionic Liquids in PVdF-co-HFP Matrices by High Pressure Infrared Spectroscopy

Teng-Hui Wang, Ming-Siou Wu and Hai-Chou Chang *

Department of Chemistry, National Dong Hwa University, Shoufeng, Hualien 974, Taiwan; 810712101@gms.ndhu.edu.tw (T.-H.W.); 610712011@gms.ndhu.edu.tw (M.-S.W.)

* Correspondence: hcchang@gms.ndhu.edu.tw; Tel.: +886-3-8903585

Figure S1. IR spectra of the [HMIM][NTF_2]/PVdF-co-HFP mixture featuring 10 wt% of [HMIM][NTf_2] obtained at (a) ambient pressure and (b) 0.4, (c) 0.7, (d) 1.1, (e) 1.5, (f) 1.8, and (g) 2.5 GPa.

Figure S2. IR spectra of the [EMIM][NTF_2]/PVdF-co-HFP mixture featuring 10 wt% of [EMIM][NTf_2] obtained at (a) ambient pressure and (b) 0.4, (c) 0.7, (d) 1.1, (e) 1.5, (f) 1.8, and (g) 2.5 GPa.

Figure S3. IR spectra of the [HMIM][NTF_2]/PVdF-co-HFP mixture featuring 50 wt% of [HMIM][NTf_2] obtained at (a) ambient pressure, (b) 2.5 GPa, and (c) cycle back to ambient pressure.

Figure S4. IR spectra of pure [EMIM][NTf_2] obtained at the time of (a) 5 min (100 scans), (b) 1 hr (1000 scans), (c) 2 hr (1000 scans), and (d) 3 hr (1000 scans) after the compression under the pressure of 0.7 GPa.

Figure S5. IR spectra of pure [EMIM][NTf_2] (pre-heated to 155^0C) obtained at (a) ambient pressure and (b) 0.4, (c) 0.7, (d) 1.1, (e) 1.5, (f) 1.8, and (g) 2.5 GPa at 25^0C.

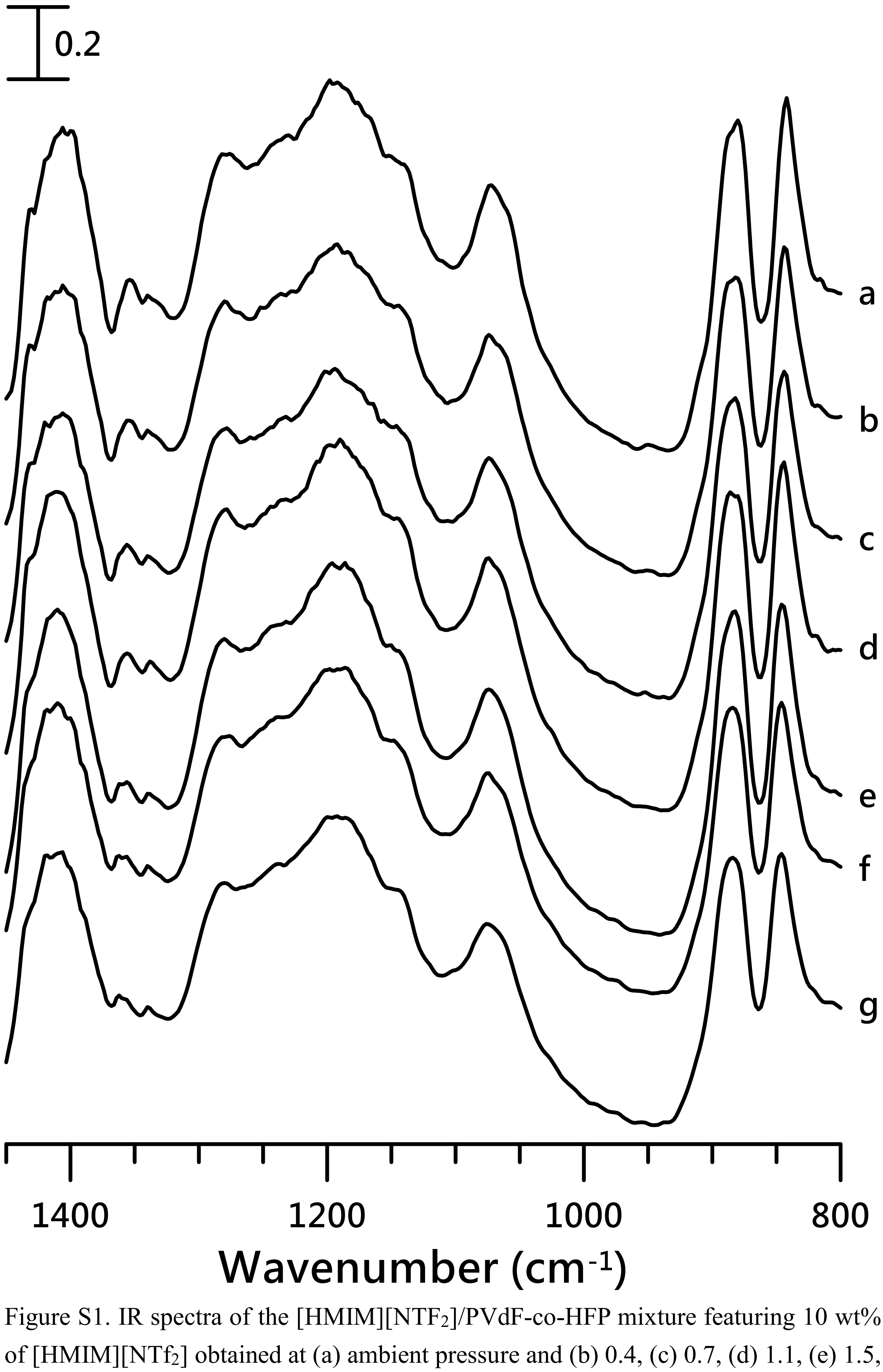

Figure S1. IR spectra of the [HMIM][NTF_2]/PVdF-co-HFP mixture featuring 10 wt% of [HMIM][NTf_2] obtained at (a) ambient pressure and (b) 0.4, (c) 0.7, (d) 1.1, (e) 1.5, (f) 1.8, and (g) 2.5 GPa.

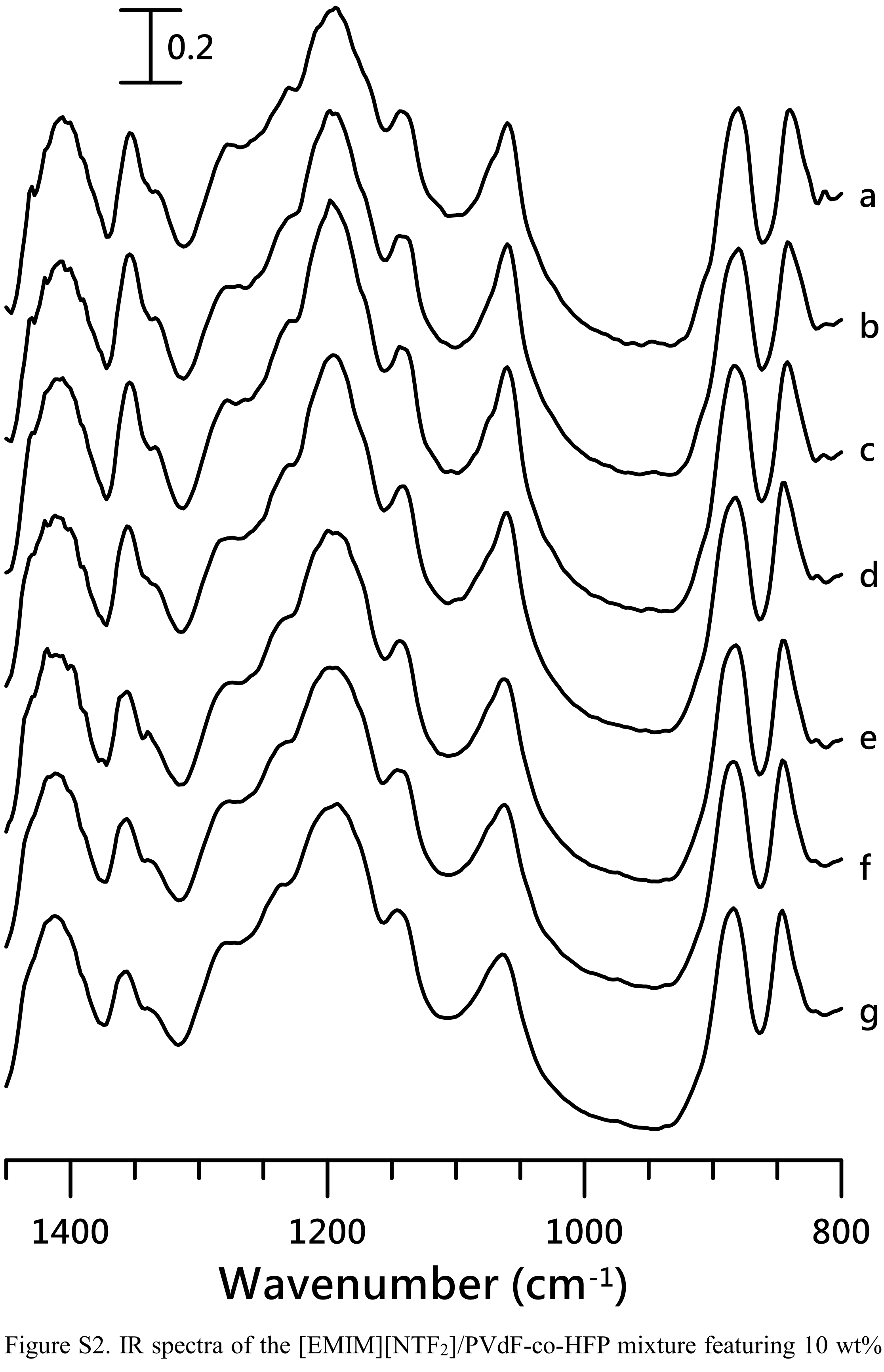

Figure S2. IR spectra of the [EMIM][NTF_2]/PVdF-co-HFP mixture featuring 10 wt% of [EMIM][NTf_2] obtained at (a) ambient pressure and (b) 0.4, (c) 0.7, (d) 1.1, (e) 1.5, (f) 1.8, and (g) 2.5 GPa.

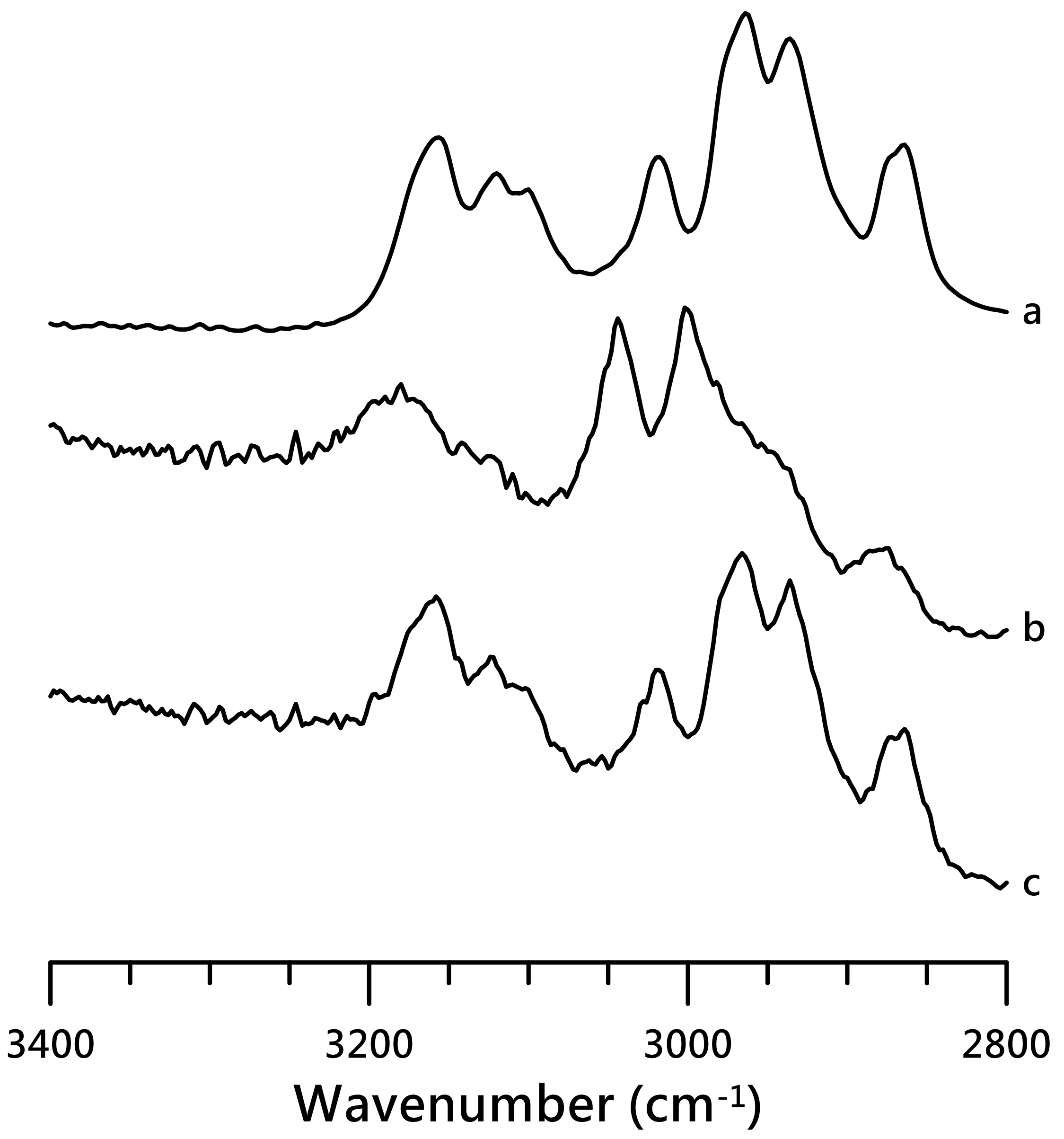

Figure S3. IR spectra of the [HMIM][NTF_2]/PVdF-co-HFP mixture featuring 50 wt% of [HMIM][NTf_2] obtained at (a) ambient pressure (in air), (b) 2.5 GPa (in DAC), and (c) cycle back to ambient pressure (in DAC).

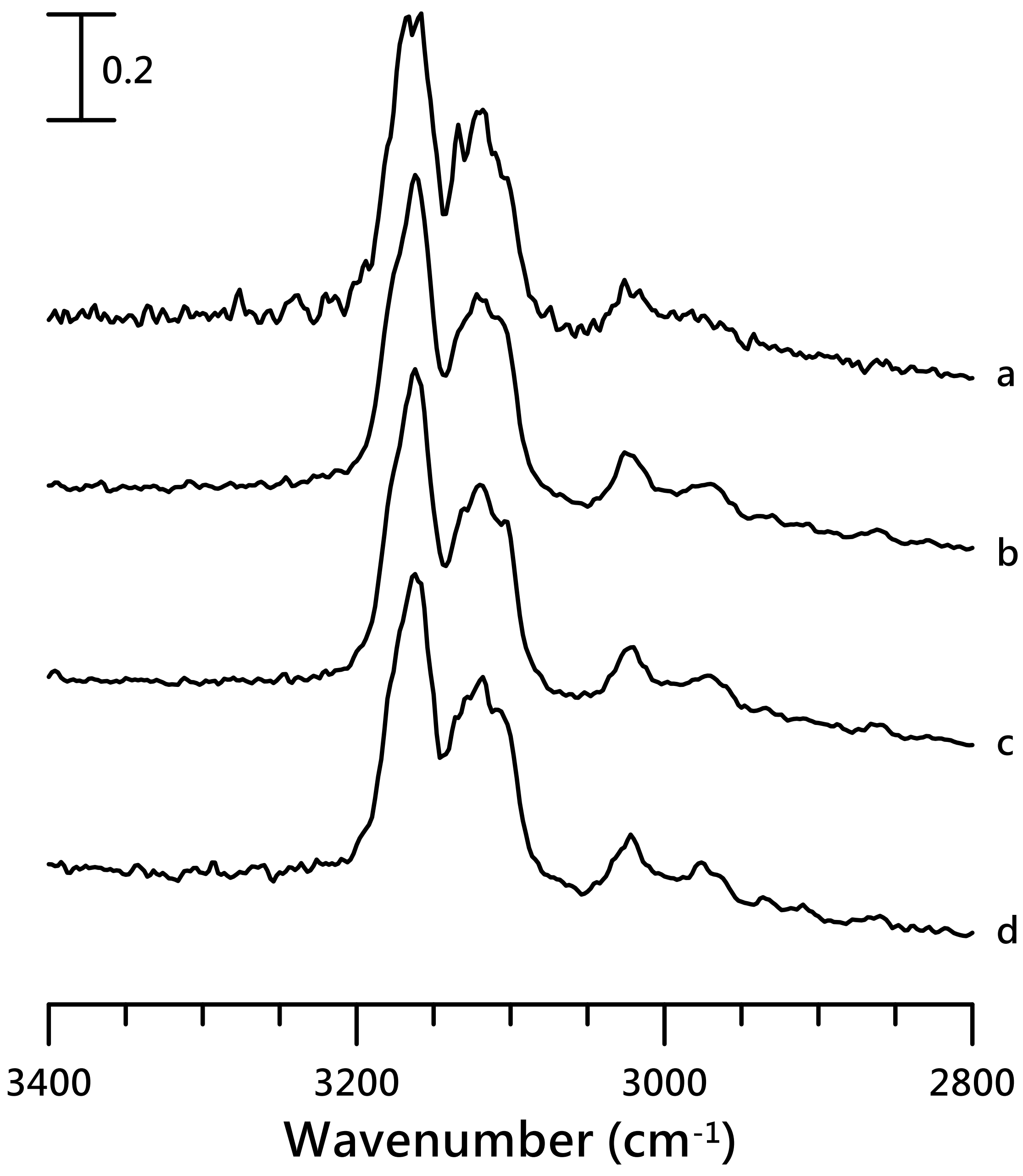

Figure S4. IR spectra of pure [EMIM][NTf_2] obtained at the time of (a) 5 min (100 scans), (b) 1 hr (1000 scans), (c) 2 hr (1000 scans), and (d) 3 hr (1000 scans) after the compression under the pressure of 0.7 GPa.

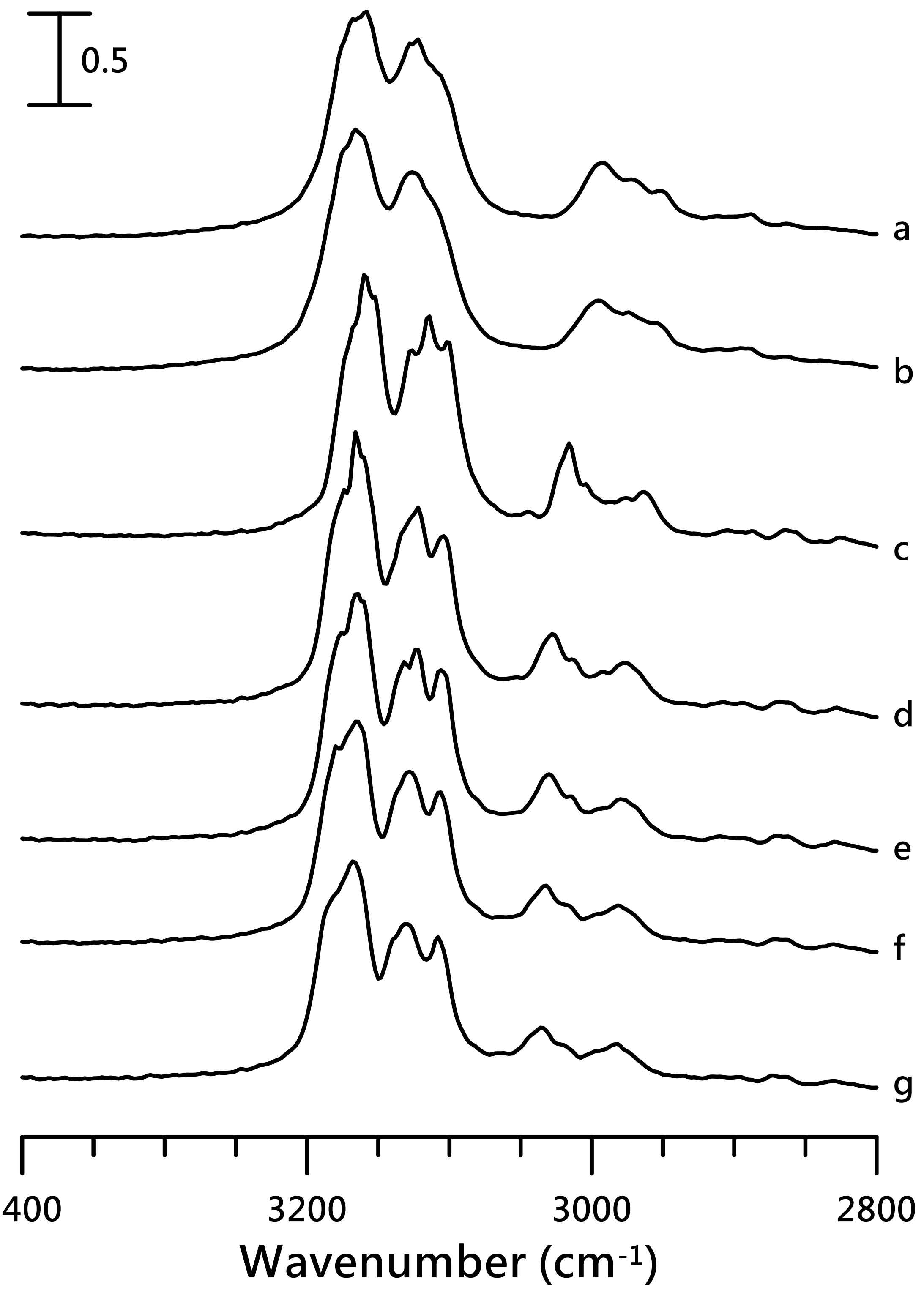

Figure S5. IR spectra of pure [EMIM][NTf_2] (pre-heated to 155^0C) obtained at (a) ambient pressure and (b) 0.4, (c) 0.7, (d) 1.1, (e) 1.5, (f) 1.8, and (g) 2.5 GPa at 25^0C.
